# Using large language models for temporal relation extraction from pediatric clinical reports

**DOI:** 10.1093/jamiaopen/ooaf121

**Published:** 2025-11-22

**Authors:** Judith Jeyafreeda Andrew, Juliette Potier, Nicolas Garcelon, Anita Burgun, Marc Vincent

**Affiliations:** Clinical Bioinformatics Laboratory, INSERM UMR1163, Imagine Institute, Université Paris Cité, Paris, F-75006, France; PRAIRIE, PaRis Artificial Intelligence Research InstitutE, PARIS, 75012, France; Clinical Bioinformatics Laboratory, INSERM UMR1163, Imagine Institute, Université Paris Cité, Paris, F-75006, France; Clinical Bioinformatics Laboratory, INSERM UMR1163, Imagine Institute, Université Paris Cité, Paris, F-75006, France; Clinical Bioinformatics Laboratory, INSERM UMR1163, Imagine Institute, Université Paris Cité, Paris, F-75006, France; PRAIRIE, PaRis Artificial Intelligence Research InstitutE, PARIS, 75012, France; Necker Enfants Malades Hospital, AP-HP, Paris, 75015, France; Clinical Bioinformatics Laboratory, INSERM UMR1163, Imagine Institute, Université Paris Cité, Paris, F-75006, France

**Keywords:** temporal relation extraction, rare diseases, patient timeline, large language model

## Abstract

**Objectives:**

To evaluate large language models (LLMs) for extracting temporal relations from pediatric rare disease clinical reports to enable automated patient timeline creation.

**Materials and Methods:**

We developed a temporal relation extraction framework for electronic health records, using 25 clinical reports from a pediatric rare disease hospital. We implemented few-shot prompting with 3 different LLMs in secure environments.

**Results:**

Our findings reveal that binary classification significantly outperforms multi-class approaches for temporal relation extraction, with best F1 scores reaching 0.70 for simpler relations while more complex relations remain challenging (F1: 0.03-0.40). Mistral 22B emerged as the strongest overall performer, though model superiority varied by relation type.

**Discussion:**

The dramatic performance improvement from reducing cognitive load (binary vs multi-class classification) demonstrates that task formulation critically impacts LLM effectiveness in specialized clinical domains. Our few-shot approach successfully enables temporal relation extraction from French pediatric texts while maintaining data privacy through local deployment, offering a viable methodology for healthcare institutions with strict data governance requirements.

**Conclusion:**

Our few-shot prompting approach demonstrated promising results in secure environments. This methodology allows technique sharing without exposing sensitive data, advancing research possibilities for clinical natural language processing in restricted settings.

## Introduction

A clinical report is a type of health record extract that conveys focused healthcare information of the patient which is prepared by or on behalf of a clinician. The research on automatic extraction of information from clinical reports has evolved greatly over time with the development of Machine Learning and Natural Language Processing (NLP) techniques. In this article, we focus on a sub-task of NLP: Temporal Relation Extraction. In particular, our end goal is to extract temporal relations from clinical reports of patients with Rare Diseases, ie, diseases that affect less than 65 out of 100 000 people worldwide.^1^ We assume that detailed histories of rare disease patients could be helpful to model the evolution of a given disease and make early diagnosis for new patients.

Although there are research works on identifying temporal relations from clinical texts, these works all focus on the detection of relations between events and temporal entities with no specific task in mind. In this paper, we focus on extracting all temporal relations between phenotypes and time entities with the aim of creating the patients timeline.

Also, while there are publicly available clinical reports^2^ for research purposes, those are mainly in the English language. The language and format of the clinical reports influences the development of techniques based on large language models (LLMs) and may result in varying performances across different languages. Another specific focus of this paper is on the French Language.

Lastly, clinical reports may vary across specialties and hospitals. The clinical reports used for our experiments are made of unstructured text and contain the history of the patient, a summary of previous visits, detailed information related to the current visit, and decisions about next visits if required. (Necker Enfants Malades Hospital, AP-HP, 149 rue de Sèvres, 75015 Paris, France. The dataset is private and cannot be distributed. The dataset is de-identified for distribution within the Institute for research purposes; however, the French regulation about health individual data prevents us from making the dataset publicly available.) This might not always be the same content in other hospitals, making validation of LLMs with local datasets essential.

Thus, in this work, we use clinical reports from patients particular to the Necker Hospital in Paris, AP-HP, 149 rue de Sèvres, 75015 Paris, France for the extraction of temporal relations. Our contributions in this paper are as follows: (i) a short description of the types of relations that have been used for a manual annotation specific to the task of automatic generation of a patient’s timeline; (ii) using 3 LLMs for temporal relation extraction to study their performance and re-usability in a secure environment; and (iii) applied to private hospital data, which are clinical reports specific to pediatric genetic rare diseases in the French language. The main results have shown that there are certain LLMs that are better in recognizing certain relation types and overall LLMs are better with binary classification than multi-class classification.

## Related work

### Annotation guidelines for relation extraction

In their study, Campillos et al^3^ present MERLOT, a French private clinical corpus comprising 500 documents annotated with 44 740 entities and 26 478 relations. The annotation involved 6 annotators over 24 months, achieving an average inter-annotator agreement of 0.793 F-measure for entities and 0.789 for relations. Pustejovsky et al^4^ present TimeML, a specification language for annotating events and temporal expressions in natural language. It introduces 4 main data structures—EVENT, TIMEX3, SIGNAL, and LINK—allowing for robust temporal anchoring and relationships among events. In the study by Andrew et al,^5^ the annotation process focuses on identifying temporal entities within clinical texts, which is crucial for building patient timelines, especially in rare diseases where data are limited. The guidelines categorize temporal entities into Dates—including Date of Visit, Date of Report, Date of Past Visit, Date of Future Visit, Date of Birth, Time, Frequency, Duration, and Age. Following this, we will present specific annotation guidelines for Relation Extraction (RE), crucial to build a patient’s timeline. Inspired from the above literature works, we identified 7 relations that could occur between phenotypes and temporal relations—BEGINS-AT, ENDS-AT, CONTAINS, OVERLAP, BEFORE, BEFORE-OVERLAP, and SIMULTANEOUS—these relation types are discussed in detail in the upcoming sections.

### Temporal relation extraction

Han et al^6^ present the OpenNRE toolkit, an open and extensible framework for neural relation extraction (RE). OpenNRE implements various neural modules and algorithms, including the BERT encoder, to support the creation of new RE models and offers typical RE models as examples for easy implementation and validation. The toolkit facilitates training custom models and quick validation, with experimental results demonstrating its effectiveness and comparability to original research implementations. Long et al^7^ present a model that combines rule-based methods with machine learning techniques, specifically Support Vector Machines and Recurrent Neural Networks, to identify TIMEX3 spans, EVENT attributes, and document-time relations. Lin et al^8^ present a BERT-based one-pass multi-task model for clinical temporal relation extraction, addressing inefficiencies in existing multi-pass methods. Raghavan et al^9^ introduce a methodology for ordering medical events in unstructured clinical narratives by learning to rank them based on their time of occurrence. Lin et al^10^ present a self-training framework that enhances Recurrent Neural Networks for temporal relation extraction in clinical texts. Lin et al^11^ also present a BERT-based approach for clinical temporal relation extraction, where instead of limiting analysis to individual sentences, the authors use a fixed window of contiguous tokens (50-60 tokens) to capture both within-sentence and cross-sentence temporal relations. Bramsen et al^12^ present a machine learning approach for temporal analysis of medical discharge summaries, focusing on temporal segmentation and ordering.

Large language models (LLMs) have been successfully used for Relation Extraction. Few-shot in-context learning entails incorporating a few training examples into model prompts, effectively “learning” via the activations induced by passing these examples through the network at inference time.^13^ The study by Wadhwa et al^14^ is one of the first attempts at using LLMs in RE. It investigates RE using GPT-3 and Flan-T5, under varying supervision levels, showing that few-shot prompting with GPT-3 achieves near state-of-the-art performance on standard RE datasets, outperforming fully supervised models. Flan-T5, while less effective in few-shot settings, achieves SOTA results when fine-tuned with chain-of-thought explanations generated by GPT-3. In this paper, we aim at using a few-shot approach to extracting temporal relations.

## Challenges in temporal relation extraction for rare diseases

Temporal relation extraction is especially challenging in rare disease contexts due to the heterogeneity and limited availability of annotated data. Unlike common conditions, rare diseases often involve complex, atypical clinical timelines that are inconsistently documented across reports. This irregularity makes it harder for models to learn robust patterns.

French clinical texts add further complexity. Temporal expressions can be subtle and vary across institutions—for example, phrases like “à l’âge de” or “depuis la première consultation” require nuanced understanding that differs significantly from English phrasing. Most existing NLP tools are trained on English datasets, so cross-lingual transfer is limited in effectiveness without domain-specific adaptation.

While there exists multilingual models, without fine-tuning on French clinical data, their performance remains inconsistent. Our approach circumvents these limitations by using few-shot prompting in French with curated examples from Necker Hospital, maintaining model privacy while ensuring linguistic and clinical relevance.

## Dataset

Thakur et al^15^ have stressed the need for external evaluation in the setting where the LLM models are to be deployed. In this work, we focus on clinical texts in the French language. This study was performed in the context of the C’IL-LICO project, a research project on ciliopathies. The C’IL-LICO project and study protocol received approval from the French National Ethics and Scientific Committee for Research, Studies and Evaluations in the Field of Health (CESREES) under the number#2201437. The data processing was approved by the French Data Protection Authority (CNIL) with a waiver of informed consent under number DR-2023-017//920398v1. Our dataset is a collection of patients’ clinical reports extracted from Dr Warehouse, the clinical data warehouse implemented at Necker Hospital.^16^ The features and capabilities of this data warehouse enable efficient use of NLP techniques in a secure environment. Following GDPR and CNIL guidelines, and as part of DrWarehouse features, the patient data are pseudonymized prior to its use in a study. The pseudonymization process is done by detecting mentions of direct identifiers in the text (name, address, phone number, hospital’s identification number, with the exception of date of birth which is relevant to the study) and replacing them with a placeholder. The detection procedure developed prior to this work in the context of DrWarehouse combines matching known identifiers from patient records, and applying machine learning models trained for Named Entity Recognition (NER) of the aforementioned identifier categories (F1 ranging from 0.95 to 1).

We have chosen 25 clinical reports for our experiments. The 25 clinical reports have been chosen such that the temporal entities, phenotypes, and relations are represented with a good amount of examples to be able to properly test the LLMs. The dataset is annotated with all-time entities and phenotypes. The time entities for the dataset are Date of Birth (DOB), Date of Report (DOR), Date of Visit (DOV), Date of Past Visit (DOPV), Date of Future Visit (DOFV), Other Dates (DOTHER), Age, Duration, Frequency, and Time. The clinical reports are pre-annotated following the short annotation guideline shown in Andrew et al.^5^ The phenotypes are also pre-annotated as per the Deep Learning technique shown in Vincent et al,^17^ where the authors use Gated Recurrent Units and Long Short Term Memory to identify phenotypes, leveraging fine-tuned word embeddings. Initially, embeddings are obtained using a skip-gram fastText model trained on a large collection of clinical reports. A second set of embeddings is created by fine-tuning a CamemBERT model, producing contextual embeddings.

In this paper, we present a short annotation guideline to annotate the temporal relation with the goal of generating a patient timeline. For the purpose of creating the prompt examples for an LLM, we choose examples from a separate set of 20 clinical reports annotated by 2 annotators, selected manually to provide prompting examples for the LLM.

**Figure 1.**
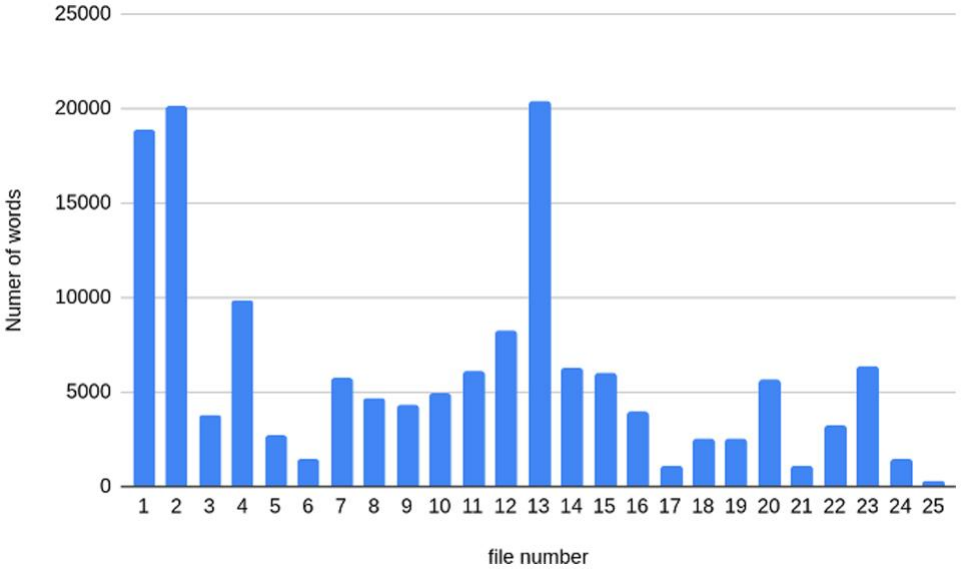
Length of each clinical report in terms of number of words.

**Figure 2.**
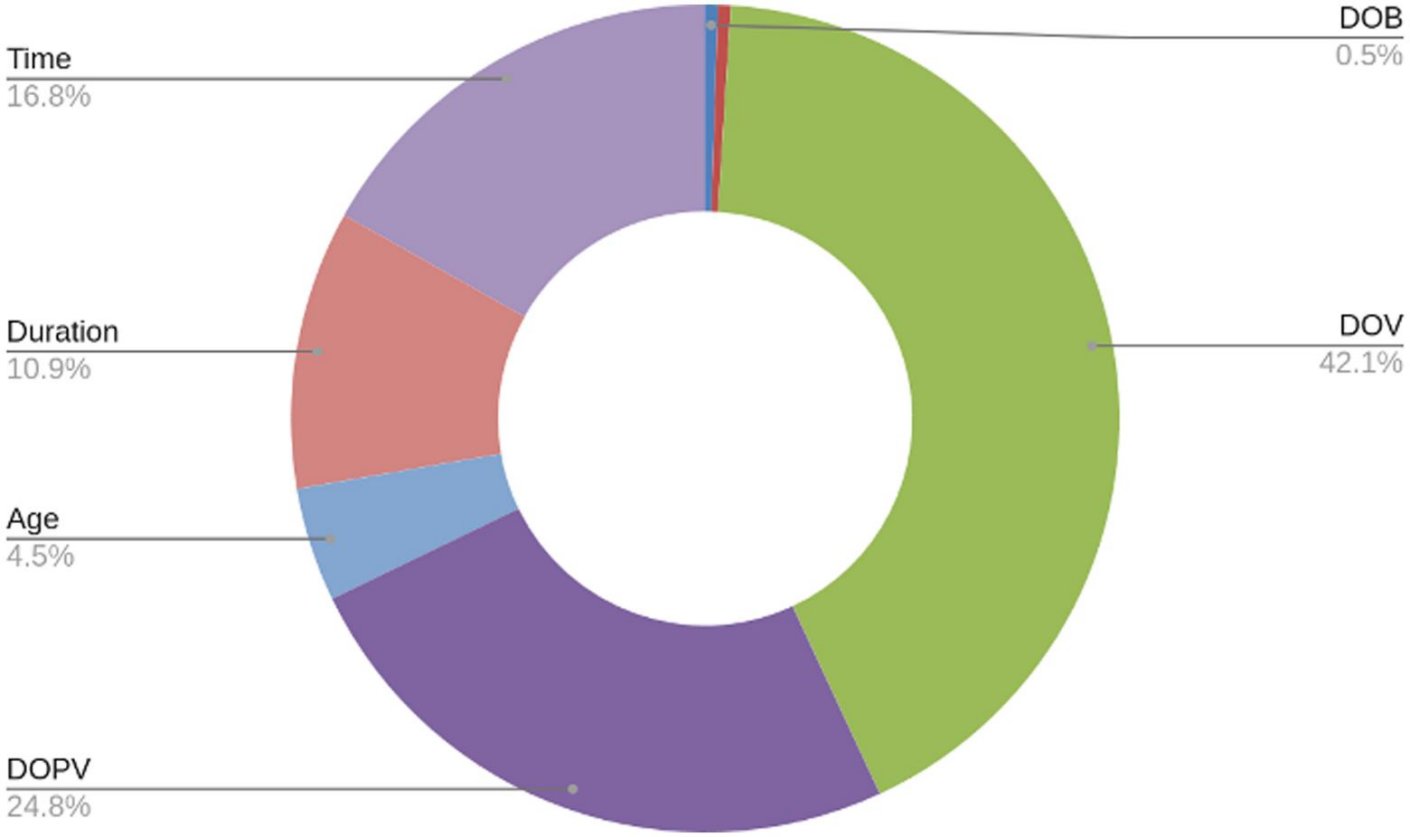
Number of relations per entity in 25 clinical reports.

The number of each temporal entity in every 1 of the 25 clinical reports used for testing the prompts is shown in a table in Appendix S1. Figure 1 shows the length of each of the 25 clinical reports, in terms of the number of words. Figure 2 and Table 1 show the number of each type of relations present between the temporal entities and phenotypes with a total of 706 relations in our test dataset of 25 clinical reports.

**Table 1.** Number of relationship types for each entity.

Ent/Rel	BEGINS-AT	ENDS-AT	CONTAINS	OVERLAP	BEFORE-OVERLAP	BEFORE	SIMULTANEOUS
DOB →“Phenotype”	1	0	1	0	7	0	0
DOR →“Phenotype”	1	0	0	0	2	0	0
DOV →“Phenotype”	85	0	7	2	181	11	0
DOPV → “Phenotype”	50	4	1	38	59	10	0
DOFV → “Phenotype”	0	0	0	0	0	0	0
DOTHER →“Phenotype”	0	0	0	0	0	0	0
Age → “Phenotype”	9	1	1	14	9	0	0
Duration → “Phenotype”	22	0	9	10	58	0	11
Frequency →“Phenotype”	0	0	0	0	0	0	3
Time → “Phenotype”	34	2	3	16	41	3	0
Total	202	7	22	80	357	24	14

The relations are in the form of “Temporal Entity” → “Phenotype.”

## Relation types

Defining temporal relations within the clinical context can be a difficult task, we build on previous works to do so, most notably the guidelines presented as part of the annotation of the MERLOT corpus.^3^ For our task of building a patient timeline, we define the following relations.

- BEGINS-AT: when a Phenotype begins on the same time point as the one referred by a time expression.
- ENDS-AT: when a Phenotype ends at the same time point as the one referred by a time expression.
- BEFORE: when a Phenotype ends before the time point referred by the time expression.
- BEFORE-OVERLAP: when a Phenotype occurs before a temporal entity and continues to occur during the timespan of the time expression.
- SIMULTANEOUS: when a Phenotype and a time entity or 2 time entities share the same time span as a time expression.
- OVERLAP: when the phenotype and temporal entity share some common time span but you do not know other information.
- CONTAINS: when a time expression contains the beginning and the end of a phenotype.

### Experimental set-up

#### Annotation process

For the purpose of testing our experiments, 2 annotators were asked to annotate (using the BRAT tool^18^) the same set of 25 clinical reports to establish a gold standard. The annotators are one linguist (J.P.) and one computer scientist (A.J.J.). The linguist is of French origin, who has done their masters in language science. The computer scientist of Indian origin with a PhD in Natural Language Processing, who has been working with clinical reports in French language for 2 years and has a B2 level of French Language. The annotations achieved an average F1 score Inter Annotator Agreement (IAA) of 0.84. The IAA for each relation type is detailed in the table in Appendix S1.

#### Large language models

For the extraction of temporal relations, we attempt a few-shot prompting approach using 3 different LLMs. For the purpose of maintaining the privacy of the data, we have used local installations of the LLMs. This allows us to maintain the privacy of the patients as no data leaves our local database. We use 3 LLMs for comparison: Llama3^19^ with 8B parameters, Gemma^20^ with 7B parameters trained on 8T tokens, and Mistral^21^ small 2409, with 22B parameters. The openai python library was used for the experiments as well as an Ollama server to privately serve the LLM models. All models were served using an 8-bit quantized format that preserves performances and lower computation requirement.

#### Prompting methods

We use 2 types of few-shot prompting where the prompts use texts tagged with phenotypes and time entities.

##### Prompt type 1: multi-class prompting

- **Goal**: Classify the relation into 1 of 7 defined temporal types (*BEGINS-AT, ENDS-AT, BEFORE, BEFORE-OVERLAP, OVERLAP, SIMULTANEOUS, CONTAINS*) or NONE.
- **Structure**:
  - Each prompt included 2 manually selected examples for every relation type, totaling 14 examples.
  - Examples were embedded within a consistent instruction block to create clear task boundaries.
  - Target entities (phenotype, time expression) were marked using HTML/XML-style tags to reduce lexical confusion and guide LLM attention.
- **Text segmentation:** 
  - For each test instance, the prompt extracted:
    - Up to 1000 tokens preceding and following the **phenotype.** 
    - Up to 1000 tokens preceding and following the **temporal entity.** 

This ensured a sufficient contextual window for LLMs while avoiding truncation.

- **Observed challenge:** 
  - Despite providing diverse examples, LLM performance was hindered, likely due to excessive cognitive load caused by choosing among 7 similar options.

##### Prompt type 2: binary classification prompting

- **Goal**: Evaluate whether a specific temporal relation exists between a phenotype and time entity—YES (relation type) or NO (NONE). In our binary classification setup, each relation type is predicted independently. We resolve cases where multiple relation types are predicted as true by selecting the one associated with the smallest training sample size. This heuristic is based on the assumption that rarer relation types are less likely to be predicted spuriously and may reflect more specific semantic patterns.
- **Structure**:
  - Separate prompt constructed for each relation type (eg one for BEGINS-AT, another for CONTAINS).
  - Included 3 positive examples and 3 NONE examples.
  - This reduces the task complexity from multi-class to binary classification.
- **Instructions simplified:** 
  - Task framed as a single decision: Does this example match the BEGINS-AT relation or not?
  - All example texts were formatted and labelled identically to maintain consistency.
- **Advantages:** 
  - Significantly higher LLM performance across all relation types.
  - Reflects cognitive load theory—simpler decisions improve model accuracy.
  - Empirically better F1 scores as demonstrated in Table 2 of our Results section.

**Design rationale:** 

- **Few-shot over fine-tuning**: Chosen to preserve data privacy and enable reproducibility on local, secure servers.
- **Manual example curation**: Ensured quality and representation for each relation despite limited annotated data.

**Table 2.** Results for multi-class and binary classification few shots prompts compared to the baseline of zero-shot and OpenNRE.

Model	BEGINS-AT	ENDS-AT	CONTAINS	BEFORE	OVERLAP	BEFORE-OVERLAP	SIMULTANEOUS
**Prompt 1 (Multi-class classification)**							
Llama	0.37	0.42	0.15	0.27	0.03	**0.51**	0.09
Mistral	**0.49**	**0.54**	0.11	**0.31**	0.11	0.50	0.15
Gemma	0.44	0.33	**0.29**	0.21	**0.25**	0.40	**0.26**
**Prompt 2 (Binary classification)**							
Llama	0.56	0.55	0.18	0.48	0.03	**0.67**	0.10
Mistral	**0.66**	**0.70**	0.17	**0.59**	0.11	0.65	0.35
Gemma	0.62	0.57	**0.40**	**0.59**	**0.37**	0.66	**0.37**
**Zero-shot prompting**							
Llama	**0.23**	**0.20**	0.10	**0.16**	**0.21**	0.17	0.09
Mistral	0.22	0.19	0.10	0.11	0.10	**0.30**	0.13
Gemma	0.18	0.16	**0.19**	0.13	**0.21**	0.17	**0.15**
**OpenNRE** ^6^							
OpenNRE^21^	0.32	0.45	0.35	0.31	0.22	0.46	0.25

The bolded values shows the model with the best performance in the category.

**Consistent formatting**: All prompts followed an identical markup and structure to reduce confusion and reinforce pattern learning.

**Figure 3.**
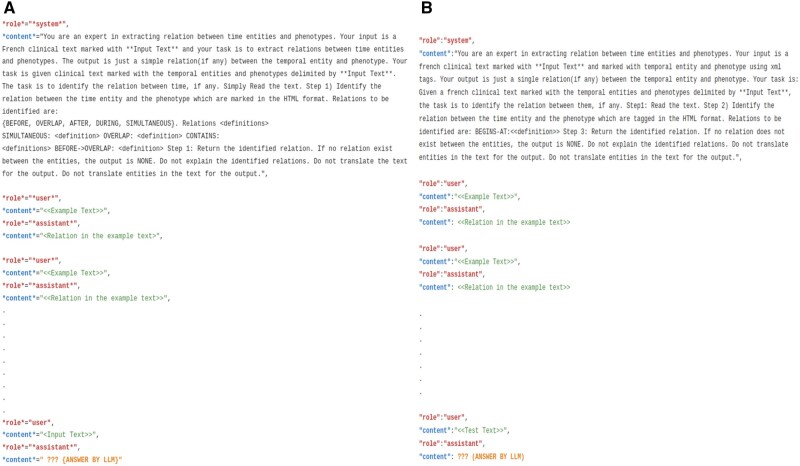
Few-shot prompting LLM approach. (A) Prompting the model to output the relation type between the given text tagged with phenotype and time entities. (B) Prompting the model for each relation type (in figure for “BEGINS-AT” given the text tagged with phenotype and time entities).

Figure 3 shows the 2 types of prompts that were used in our experiments.

## Results and analysis

Results achieved by the 3 LLM with 2 types of few-shot prompts are displayed in Table 2. Table 2 also shows the results of binary zero-shot classification and OpenNRE. For binary zero shot classification, we have used similar prompt as prompt 2 without any example, ie the LLMs are given a definition of a single relation type and are asked to classify the input text into 1 of the 2 classes (Relation typed define or NONE); however, there are no previous examples given to demonstrate this relation type to the. LLM. Table 2 clearly shows that the LLM can learn from few representative examples. While the LLM is prompted to identify the relation given several choices, the tested LLMs does not seem to perform very well leading us to believe that they tend to get confused given many choices. On the other hand, while we used the second prompt, the performance of LLM increases, confirming that multiple choices and complex instructions might hinder performances w.r.t a simpler formulation of the problem. It can be seen that for each relation type there is a different LLM that performs better than the other. On an average, it can be argued that Gemma performs better than the other 2 LLM as it has significantly better results for CONTAINS and OVERLAP. However, from this table, we realized that the “OVERLAP,” “CONTAINS,” and “SIMULTANEOUS” relations are complicated relations to capture for an LLM. This could be due to one of the following factors: (i) Not enough examples were used for the few-shot approach. This is seen specifically in the case of SIMULTANEOUS as the number of this relation type is few when compared to the others. (ii) The tested LLMs confuse the definition for the relation. This is seen specifically in the case of BEFORE-OVERLAP and OVERLAP that have similar definitions but can be differentiated better only with the context. (iii) Not enough context is available for the LLM to understand the relation type, causing confusions between similar relation types such as BEFORE, BEFORE-OVERLAP, and OVERLAP.

### Comparative evaluation

To contextualize the performance of our few-shot prompting framework, we included comparisons with 2 baseline methods: zero-shot prompting and the OpenNRE toolkit.^21^ Zero-shot prompting involves providing only a relation definition, with no examples included. The LLMs were asked to determine whether a specific relation (eg BEGINS-AT) exists between a marked phenotype and time entity. This minimalist setup tests the models’ general language understanding without domain-specific guidance. We also evaluated the performance of the OpenNRE framework,^6^ which offers pre-implemented neural architectures for relation extraction, including BERT-based models.

### Performance by approach

**Binary Classification** consistently outperforms both multi-class classification and zero-shot approaches across all models and relation types. The average improvement from multi-class to binary classification is approximately 46% (from 0.31 to 0.55 average F1), demonstrating that cognitive load reduction significantly enhances LLM performance in temporal relation extraction.

**Multi-class Classification** shows moderate performance, with models struggling to distinguish between 7 different relation types simultaneously. This aligns with cognitive load theory, where too many simultaneous choices overwhelm the model’s processing capacity.

**Zero-shot Classification** demonstrates the poorest performance, confirming that few-shot learning with examples is crucial for this specialized clinical task. Interestingly, the traditional OpenNRE baseline often outperforms zero-shot LLMs, suggesting that domain-specific training remains valuable.

**OpenNRE** surpassed zero-shot LLMs across all relation types. For example, CONTAINS yielded an F1 score of **0.35**, and BEFORE-OVERLAP reached **0.46**—both higher than any zero-shot LLM performance.

### Interpretation

These comparative results demonstrate that:

- **Few-shot prompting outperforms both baselines**, especially when binary classification reduces cognitive load.
- **Zero-shot LLM prompting is inadequate** for temporal RE without examples.
- **OpenNRE provides a useful baseline**, but lacks the adaptability and privacy-preserving deployment offered by LLMs in our setup.

### Model rankings by weighted performance

- Mistral (22B): Best overall performer with average F1 of 0.55 (binary classification)
- Gemma (7B): Competitive at 0.54 average, with superior performance on complex relations
- Llama3 (8B): Underperforms at 0.45 average across relations

The results reveal a bimodal distribution of relation extraction difficulty:

**High-performing relations:** BEGINS-AT, ENDS-AT, BEFORE-OVERLAP (F1: 0.55-0.70).

**Low-performing relations:** OVERLAP, CONTAINS, SIMULTANEOUS (F1: 0.03-0.40).

It should also be noted that even with perfect human precision (table in Appendix S1) LLMs struggle significantly, with the best binary classification results (Table 2) still showing substantial gaps compared to human performance. For example, SIMULTANEOUS relations have 0.89 human F1 but only 0.37 best LLM F1.

The improvement from multi-class to binary prompting demonstrates cognitive load theory in action—when LLMs must choose between 7 relations simultaneously, performance degrades dramatically, similar to human working memory limitations.

This gap suggests fundamental differences in how LLMs process temporal semantics, with clear temporal boundaries being more tractable than fuzzy or complex temporal relationships.

## Research context and theoretical implications temporal reasoning in clinical contexts

**Cognitive Load Theory Application:** The dramatic performance difference between binary (Prompt 2) and multi-class (Prompt 1) classification aligns with cognitive load theory. When presented with 7 simultaneous classification options, LLMs exhibit similar limitations to human cognitive processing—the working memory becomes overwhelmed, leading to degraded performance.

**Linguistic Complexity in Medical French:** The study’s focus on French clinical texts generated by medical doctors without any constraint, to deliver information about rare disease patients’ medical histories, introduces additional complexity layers, such as:

- Medical terminology variability: French medical language has multiple ways to express temporal relationships
- Syntactic structures: French temporal expressions often involve complex prepositional phrases that may confuse LLMs
- Cultural medical reporting styles: French clinical reports may have different narrative structures compared to English texts used in LLM training

**Few-Shot Learning Limitations:** With only 2-3 examples per relation type in few-shot prompts, the study reveals fundamental limitations in LLM sample efficiency for specialized domains. Thus, complex relations like OVERLAP may require more diverse examples. Furthermore, clinical temporal relations are highly context-dependent, requiring more examples to capture variability. Some relations (SIMULTANEOUS: F1 = 0.09-0.37) may be inherently rare, making few-shot learning ineffective.

## Conclusion

In this paper, we have presented an analysis of using LLMs for the task of Relation extraction using 2 formats of few-shot prompts to understand the capabilities of each tested LLMs, showing that a simpler task formulation corresponding to binary classification yields better results. In this work, we focused on LLMs as our motivation was to deploy/share models that can extract information without giving away data (which, given no information is given in the request prompts, an LLM can do). With this goal in mind, the 25 reports we described here have presented us with a good number of relation types between entities so as to enable us to learn more about using LLMs for the Relation Extraction Task. Comparisons of the provided method to BERT based baselines will be made possible in future works by extending the dataset in order to allow fine tuning of said models.

## Supplementary Material

ooaf121_Supplementary_Data

## Data Availability

Due to the highly specific nature of the text data used in this study, and the practical impossibility of guaranteeing complete anonymization at the individual level, French regulations do not allow us to publicly share these data outside the framework of the research agreement, the designated research partners, and the scope of patient consent. Aggregate data, however, are available upon request to the authors. Prior to sharing, a data use agreement must be negotiated between the 2 parties in order to comply with applicable guidelines and regulations. In accordance with these regulations,^22^ the dataset will be archived and stored in a secure enclave. Outside the scope of the current research, access to patient data may be granted only upon submission of a research project to a duly authorized scientific and ethics committee. The process can be initiated by contacting dpd@institutimagine.org.
