# Supplementary material for: Using large language models for temporal relation extraction from pediatric clinical reports
Source: JAMIA Open. 2025 Nov 22;8(6):ooaf121. doi: 10.1093/jamiaopen/ooaf121 (PMC12640238; doi:10.1093/jamiaopen/ooaf121)
Supplement: ooaf121_Supplementary_Data [file ooaf121_supplementary_data.docx]

**ANNEXE:**

**Prompt from Figure 2a:**

*role*="*system*",

*content*="You are an expert in extracting relation between time entities and phenotypes. Your input is a French clinical text marked with **Input Text** and your task is to extract relations between time entities and phenotypes. The output is just a simple relation(if any) between the temporal entity and phenotype. Your task is given clinical text marked with the temporal entities and phenotypes delimited by **Input Text**. The task is to identify the relation between time, if any. Simply Read the text. Step 1) Identify the relation between the time entity and the phenotype which are marked in the HTML format. Relations to be identified are:

{BEFORE, OVERLAP, AFTER, DURING, SIMULTANEOUS}. Relations <definitions>

SIMULTANEOUS: <definition> OVERLAP: <definition> CONTAINS:

<definitions> BEFORE->OVERLAP: <definition> Step 1: Return the identified relation. If no relation exists between the entities, the output is NONE. Do not explain the identified relations. Do not translate the text for the output. Do not translate entities in the text for the output.",

*role*="*user*",

*content*="<<Example Text>>,

*role*="*assistant*",

*content*="<Relation in the example text>",

*role*="*user*",

*content*="<<Example Text>>,

*role*="*assistant*",

*content*="<<Relation in the example text>>",

.

.

.

.

.

.

.

.

.

*role*="user",

*content*="<Input Text>>,

*role*="*assistant*",

*content*=" ??? {ANSWER BY LLM}

**Prompt from FIgure 2b**

"role":"system",

"content":"You are an expert in extracting relation between time entities and phenotypes. Your input is a french clinical text marked with **Input Text** and marked with temporal entity and phenotype using xml tags. Your output is just a single relation(if any) between the temporal entity and phenotype. Your task is: Given a french clinical text marked with the temporal entities and phenotypes delimited by **Input Text**, the task is to identify the relation between them, if any. Step1: Read the text. Step 2) Identify the relation between the time entity and the phenotype which are tagged in the HTML format. Relations to be identified are: BEGINS-AT:<<definition>> Step 3: Return the identified relation. If a relation does not exist between the entities, the output is NONE. Do not explain the identified relations. Do not translate entities in the text for the output. Do not translate entities in the text for the output.",

"role":"user",

"content":"<<Example Text>>",

"role":"assistant",

"content": <<Relation in the example text>>

"role":"user",

"content":"<<Example Text>>",

"role":"assistant",

"content": <<Relation in the example text>>

.

.

.

.

.

.

.

"role":"user",

"content":"<<Test Text>>",

"role":"assistant",

"content": ??? (ANSWER BY LLM)

**Tables:**

| File number | DOB | DOR | DOV | DOPV | DOFV | DOTHER | AGE | DURATION | FREQUENCY | TIME |
| --- | --- | --- | --- | --- | --- | --- | --- | --- | --- | --- |
| 1 | 7 | 8 | 5 | 64 | 9 | 56 | 5 | 25 | 26 | 17 |
| 2 | 1 | 0 | 2 | 3 | 0 | 7 | 2 | 5 | 0 | 5 |
| 3 | 9 | 10 | 2 | 18 | 2 | 5 | 7 | 10 | 38 | 9 |
| 4 | 2 | 1 | 1 | 0 | 0 | 4 | 2 | 4 | 0 | 1 |
| 5 | 2 | 2 | 4 | 0 | 0 | 1 | 0 | 1 | 6 | 4 |
| 6 | 3 | 3 | 1 | 1 | 2 | 1 | 1 | 1 | 1 | 3 |
| 7 | 0 | 0 | 0 | 1 | 0 | 1 | 0 | 0 | 0 | 0 |
| 8 | 11 | 5 | 14 | 18 | 0 | 15 | 8 | 20 | 30 | 108 |
| 9 | 1 | 3 | 1 | 1 | 0 | 0 | 0 | 0 | 0 | 0 |
| 10 | 1 | 5 | 3 | 7 | 2 | 8 | 4 | 11 | 22 | 9 |
| 11 | 3 | 3 | 3 | 1 | 0 | 7 | 1 | 3 | 5 | 6 |
| 12 | 1 | 3 | 3 | 3 | 0 | 1 | 1 | 4 | 2 | 4 |
| 13 | 1 | 1 | 2 | 0 | 0 | 0 | 3 | 0 | 2 | 2 |
| 14 | 2 | 1 | 2 | 0 | 0 | 2 | 0 | 2 | 2 | 1 |
| 15 | 0 | 1 | 2 | 0 | 0 | 0 | 0 | 1 | 9 | 0 |
| 16 | 1 | 2 | 1 | 2 | 3 | 1 | 1 | 0 | 2 | 4 |
| 17 | 1 | 3 | 4 | 3 | 0 | 2 | 1 | 1 | 0 | 3 |
| 18 | 2 | 2 | 2 | 3 | 1 | 2 | 2 | 3 | 9 | 10 |
| 19 | 1 | 2 | 1 | 0 | 0 | 1 | 1 | 1 | 0 | 0 |
| 20 | 1 | 4 | 4 | 4 | 1 | 9 | 1 | 2 | 12 | 8 |
| 21 | 1 | 1 | 3 | 1 | 1 | 12 | 3 | 6 | 13 | 2 |
| 22 | 0 | 0 | 0 | 0 | 0 | 0 | 0 | 0 | 0 | 4 |
| 23 | 2 | 1 | 1 | 4 | 0 | 0 | 1 | 0 | 0 | 4 |
| 24 | 0 | 0 | 0 | 0 | 0 | 0 | 0 | 0 | 0 | 1 |
| 25 | 1 | 1 | 0 | 0 | 0 | 0 | 0 | 0 | 0 | 1 |

**Table : Number of each entity in every one of the 25 files**

| **RELATION** | **PRECISION** | **RECALL** | **F1** |
| --- | --- | --- | --- |
| BEGINS-AT | 1 | 0.8 | 0.8 |
| ENDS-AT | 1 | 1 | 1 |
| CONTAINS | 1 | 0.7 | 0.83 |
| BEFORE | 1 | 0.67 | 0.8 |
| OVERLAP | 1 | 0.49 | 0.66 |
| BEFORE-OVERLAP | 1 | 0.7 | 0.82 |
| SIMULTANEOUS | 1 | 0.8 | 0.89 |

**Table : Inter Annotator Agreement(IAA) for each relation type**

**AGGREGATE DATA ABOUT THE CORPUS:**

Regarding medical information, due to the GDPR law and the risk of re-identifying patients in the rare disease domain, we are unable to provide individual patient information irrespective of the corpus being de-identified. Thus we can only provide aggregate data.

The corpus corresponds to 25 distinct patient files. A total of 615 phenotypes have been studied.

The patients have a mean age of 6 years at the time of admission, max age of 12 years and 4 months and min age of 10 months

Gender was explicitly mentioned in text in 19 files out of 25, corresponding to 11 Male patients and 8 Female patients. The radiology and test reports in the corpus do not have any gender mentioned.

The ethnicity of any patient is not disclosed in the text. It is to be noted that all files are from the Necker Hospital, Paris, France^1^
